# Supplementary material for: Exploring Compassion towards Laboratory Animals in UK- and China-Based Undergraduate Biomedical Sciences Students
Source: Animals (Basel). 2023 Nov 20;13(22):3584. doi: 10.3390/ani13223584 (PMC10668705; doi:10.3390/ani13223584)
Supplement: Supplementary file 1 [file animals-13-03584-s001.zip › animals-2688663-supplementary.pdf]

Supplementary material

Laboratory Animal Compassion Survey (LACS)

Compassion towards laboratory animals. Participants were asked to indicate to what extent they agreed or disagreed with the following statements. The answer options were Strongly Agree, Agree, Slightly Agree, Slightly Disagree, Disagree or Strongly Disagree; there was no neutral answer option. For 'pro-compassion' statements the answer options were scored 5 to 0 respectively, i.e., 'Strongly Agree' scored 5. The 'anti-compassion' statements were reverse scored, i.e., 'Strongly Agree' scored 0. The total score (range 0-60) comprised the equally-weighted sum of the score for each question.

Pro-compassion statements:

- It is morally wrong to cause suffering in laboratory rodents. ("Q1" in Supplementary Figure 2)
- Laboratory rodents should be allowed to live in an environment that allows them to express natural behaviours. For example, social interactions, building nests and digging. ("Q3")
- I get upset, or believe I would get upset, if I cause suffering to laboratory rodents as part of a scientific experiment. For example, by withholding food for several days or injecting a substance into a non-anesthetised animal. ("Q4")
- The laws that regulate scientific work with laboratory rodents help biomedical scientists carry out their research. ("Q7")
- Laboratory rodents that are not needed or used for experimental purposes must be killed in the most humane way possible. ("Q8")

- I enjoy, or I believe I would enjoy, caring for laboratory rodents. For example, handling them, feeding them, and ensuring their living environment was properly maintained. (“Q11”)

Anti-compassion statements:

- Biomedical scientists have the right to use laboratory rodents as they wish. (“Q2”)
- Using laboratory rodents in practical classes is essential when studying for an undergraduate biomedical sciences degree. (“Q5”)
- It is acceptable to breed very large numbers of laboratory rodents even if the majority of these will not be used directly in experiments. (“Q6”)
- I think of laboratory rodents mainly as a research tool. (“Q9”)
- It is acceptable to create a genetically modified laboratory rodents to be used in research, even if the genetic modification causes these animals to suffer. (“Q10”)
- Laboratory rodents are not capable of expressing emotion in a way that can be detected by biomedical researchers. (“Q12”)

Text used for self-reporting level of experience of working with lab animals. “Please indicate your highest level of experience of working with laboratory animals during your undergraduate degree programme. Your experience may involve direct or indirect contact with laboratory animals. Direct contact could be, for example, helping to carry out a behavioural experiment or collecting biological material from a euthanised animal. Indirect contact could be, for example, using tissue from an animal in a laboratory-based practical class. Select a value between 1-5, where 1 equates to no experience at all, and 5 equates to a relatively high level of experience. For example, a student who uses animals in in vivo experiments in their Year 4 project would likely select 5; a student who has used animal tissues (prepared by someone else) in a laboratory-based practical class would likely select 2.”

Text used for harm-benefit analysis section. "In this section, we want you to imagine you are designing a research programme. Each programme uses the same (and scientifically appropriate) number of laboratory rats. For each programme, please choose what level of suffering you feel is acceptable in order to achieve the scientific outcomes of the research study.

Research programme 1 will provide a simple, effective and safe treatment that will prevent the onset of Type 2 diabetes in individuals with a risk of developing this disease. Type 2 diabetes is a metabolic disease that affects ~350 million people globally, has a strong and negative day-to-day impact on health, and results in the death of ~3 million people every year.

Research programme 2 will result in a simple, effective and safe treatment that will prevent the onset of fibrodysplasia ossificans progressiva (FOP) in individuals with a risk of developing this disease. FOP affects a total of ~3,000 people worldwide. This disease is characterized by the transformation of skeletal muscle, tendons, ligaments, and other tissues into bone. People with FOP are usually unable to move before the age of 20 and die at a median age of 40.

Research programme 3 will provide a simple, effective and safe treatment that will prevent the onset of psoriasis. Psoriasis is a chronic immunological/dermatological disease that results in small patches of red and itchy skin, normally at the elbows and knees. Psoriasis affects ~150 million people globally. There is currently no cure, but it can be readily treated symptomatically. There is no strong scientific evidence showing that people with psoriasis have a different mortality rate compared to people who do not have psoriasis.

Answer options were: No deliberate suffering at all/Mild suffering/Moderate suffering/Severe suffering.

Questions on consciousness and emotion in humans and other animals:

1. How certain are you that you (the person completing this survey) are conscious?

Answer options: I am certain I am conscious / I am fairly sure I am conscious / I think it is possible that I am conscious / I don’t know whether or not I am conscious / I think it is unlikely that I am conscious / I am fairly sure I am not conscious / I am certain I am not conscious / It is impossible for me to know whether or not I am conscious

2. How certain are you that you experience emotions?

Answer options: I am certain I experience emotions / I am fairly sure I experience emotions / I think it is possible that I experience emotions / I don’t know whether or not I experience emotions / I think it is unlikely that I experience emotions / I am fairly sure I do not experience emotions / I am certain I do not experience emotions / It is impossible for me to know whether or not I experience emotions

3. How certain are you that other humans are conscious?

Answer options: I am certain that other humans are conscious / I am fairly sure other humans are conscious / I think it is possible that other humans are conscious / I don’t know whether or not other humans are conscious / I think it is unlikely that other humans are conscious / I am fairly sure other humans are not conscious / I am certain other humans are not conscious / It is impossible for me to know whether or not other humans are conscious

4. How certain are you that other humans experience emotions?

Answer options: I am certain that other humans experience emotions / I am fairly sure that other humans experience emotions / I think it is possible that other humans experience emotions / I don’t know whether or not other humans experience emotions / I think it is unlikely that other humans experience emotions / I am fairly sure that other humans do not experience emotions / I am certain that other humans do not experience emotions / It is impossible for me to know whether or not other humans experience emotions

5. Several species of rodents, including mice (*Mus musculus*) and rats (*Rattus norvegicus domestica*), are commonly used in biomedical research. Taking the rat as an example, how certain are you that rats are conscious?

Answer options: I am certain that rats are conscious / I am fairly sure rats are conscious / I think it is possible that rats are conscious / I don't know whether or not rats are conscious / I think it is unlikely that rats are conscious / I am fairly sure rats are not conscious/ I am certain rats are not conscious / It is impossible for me to know whether or not rats are conscious

6. How certain are you that rats experience emotions?

Answer options: I am certain that rats experience emotions / I am fairly sure that rats experience emotions / I think it is possible that rats experience emotions / I don't know whether or not rats experience emotions / I think it is unlikely that rats experience emotions / I am fairly sure that rats do not experience emotions / I am certain that rats do not experience emotions / It is impossible for me to know whether or not rats experience emotions

7. Several other non-mammalian species, including fruit flies (*Drosophila melanogaster*) and nematode worms (*Caenorhabditis elegans*), are also commonly used in biomedical research. Taking the fruit fly as an example, how certain are you that fruit flies are conscious?

Answer options: I am certain that fruit flies are conscious / I am fairly sure fruit flies are conscious / I think it is possible that fruit flies are conscious / I don't know whether or not fruit flies are conscious I think it is unlikely that fruit flies are conscious / I am fairly sure fruit flies are not conscious / I am certain fruit flies are not conscious / It is impossible for me to know whether or not fruit flies are conscious

8. How certain are you that fruit flies experience emotions?

Answer options: I am certain that fruit flies experience emotions / I am fairly sure that fruit flies experience emotions / I think it is possible that fruit flies experience emotions / I don't know whether or not fruit flies experience emotions / I think it is unlikely that fruit flies experience emotions / I am fairly sure that fruit flies do not experience emotions / I am certain that fruit flies do not experience emotions / It is impossible for me to know whether or not fruit flies experience emotions

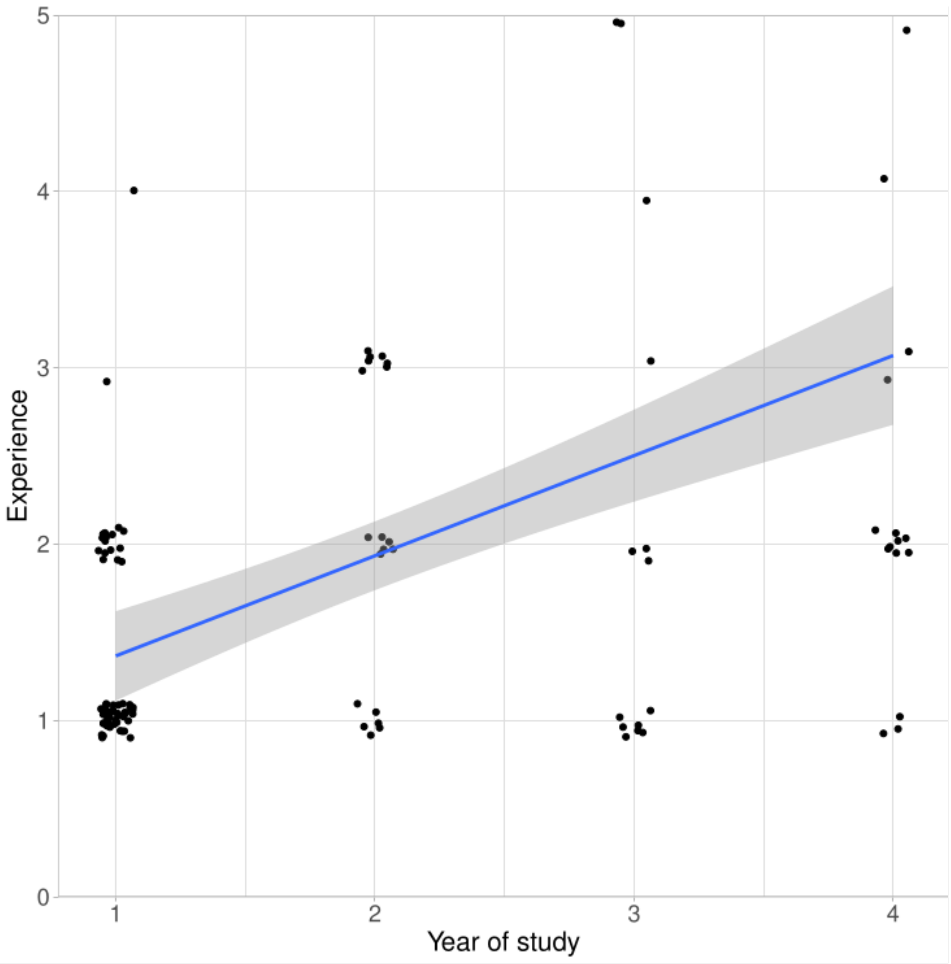

Supplementary Figure S1. Level of experience correlates strongly with year of study. The blue line shows linear fit, grey bands represent 95% confidence intervals.

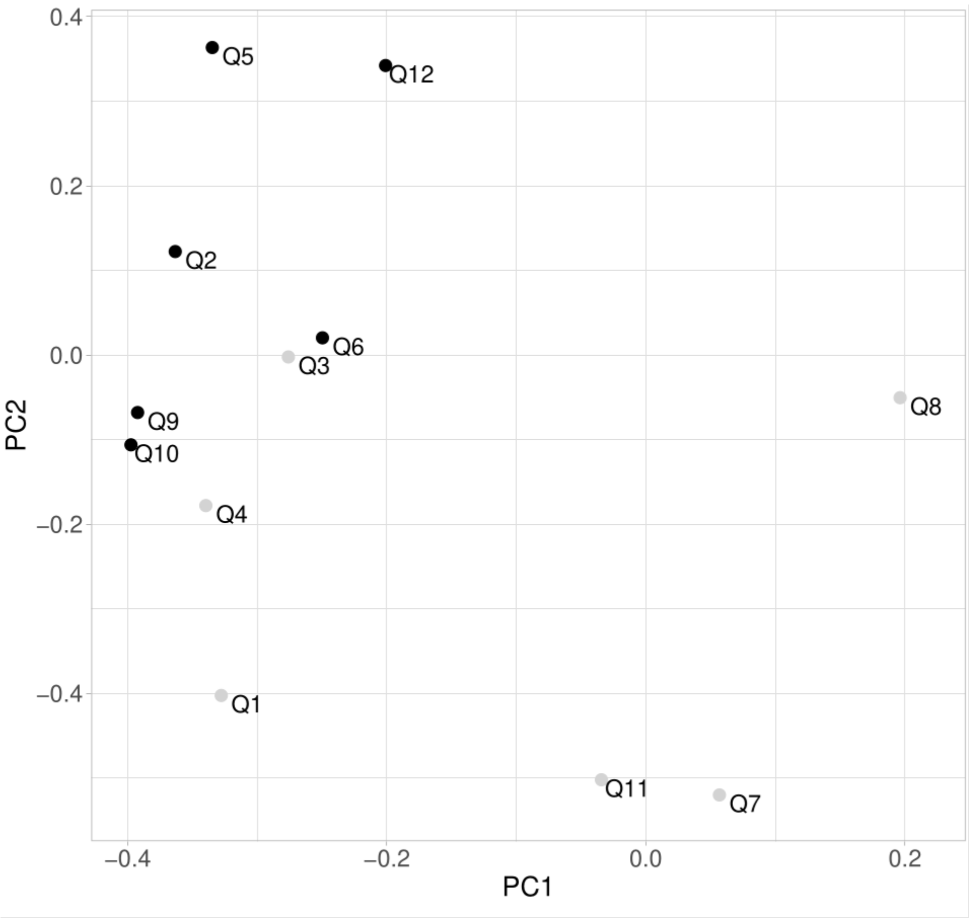

Supplementary Figure S2. Validation of survey items. Principal component analysis shows clustering of pro- (grey) or anti-compassion (black) statements.

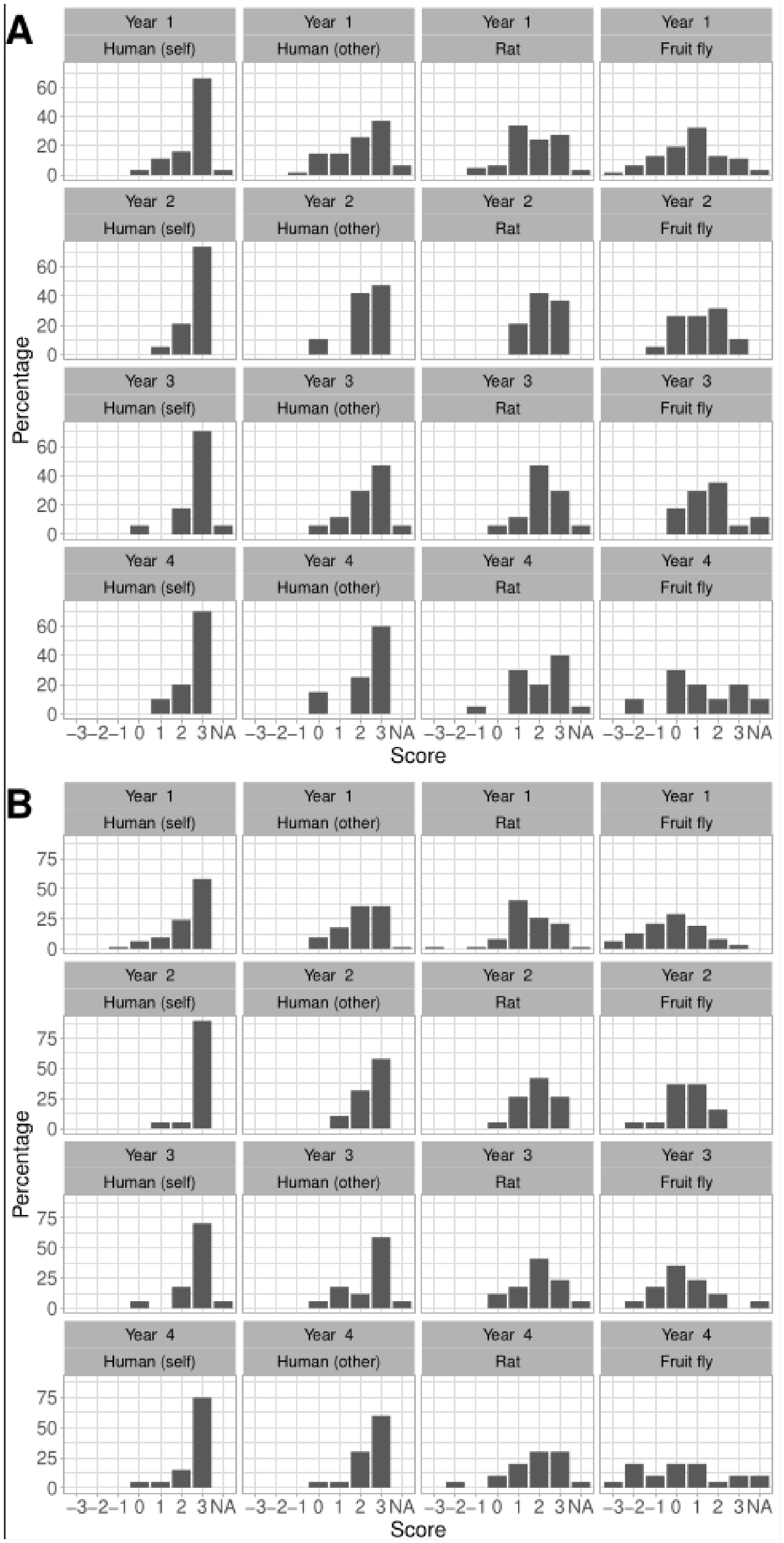

Supplementary Figure S3. Attribution of (A) consciousness and (B) emotions to self, other humans, rats and fruit flies by year of study.

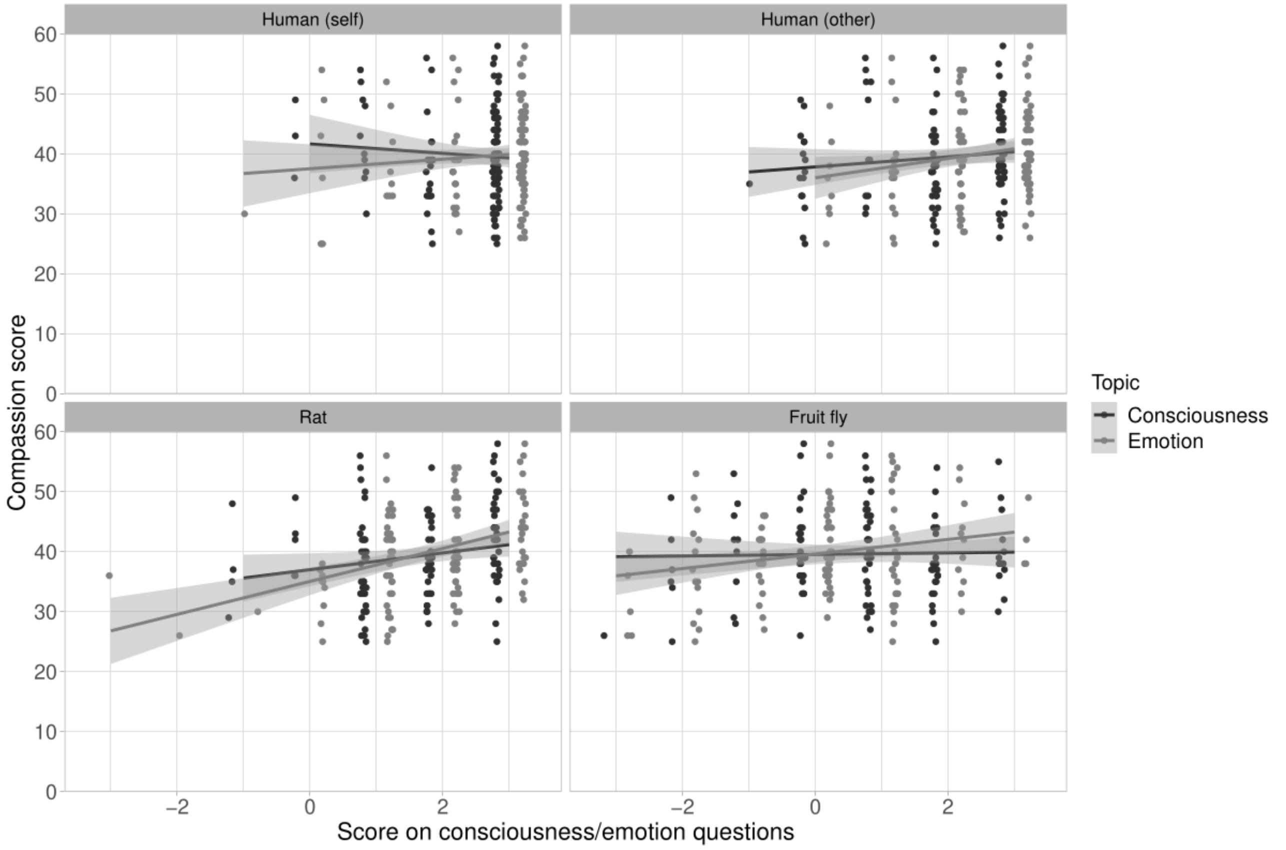

Supplementary Figure S4. Correlations between beliefs about consciousness/emotion and compassion scores. The lines show linear fit, grey bands represent 95% confidence intervals.

Supplementary Table S1. Correlations between beliefs about consciousness or emotion and compassion scores.

Species	Pearson's R	Adjusted p value
**Consciousness**		
**Self**	-0.079	0.615
**Other humans**	0.123	0.195
**Rat**	0.198	0.034
**Fruit fly**	0.024	0.798
**Emotion**		
**Self**	0.095	0.615
**Other humans**	0.200	0.063
**Rat**	0.405	1.41×10^{-5}
**Fruit fly**	0.228	0.029
